# Supplementary material for: Large carnivore habitat suitability modelling for Romania and associated predictions for protected areas
Source: PeerJ. 2019 Mar 19;7:e6549. doi: 10.7717/peerj.6549 (PMC6430102; doi:10.7717/peerj.6549)
Supplement: Supplemental Information 1 [file peerj-07-6549-s001.docx]

**Supplementary material**

***** Cristescu B, Domokos C, Teichman KJ, Nielsen SE. Large carnivore habitat suitability modelling for Romania and associated predictions for protected areas. *PeerJ*.

* Author for correspondence:

cristesc@ualberta.ca

Table S1. Land cover categories used in modelling large carnivore occurrence in Romania based on Corine land cover reclassification (EIONET, 2013). Bare rock, beaches, dunes and sand as well as peat bogs were not included as they were not hypothesized to be conducive of carnivore occurrence.

| **Original Corine land cover** | **Reclassification for modelling procedure** |
| --- | --- |
| Broadleaved forest | Broadleaf forest |
| Mixed forest | Mixed forest |
| Conifer forest | Conifer forest |
| Scrub and/or herbaceous vegetation association (moors and heathland; natural grasslands; transitional woodland shrub) | Shrub/Herbaceous |
| Agriculture (complex cultivation patterns; fruit trees and berry plantations; land principally occupied agric signif areas nat veget; non-irrigated arable land; rice fields; vineyards) | Cultivation |
| Agriculture (pastures) | Pasture |
| Artificial surfaces (airports; construction sites; discontinuous urban fabric; dump sites; green urban areas; industrial or commercial units; mineral extraction sites; port areas; road and rail network and associated land; sport and leisure facilities) | Artificial |

Table S2. Candidate model set for brown bear occurrence in Romania. Variable codes listed under ‘Model structure’ are provided in Table 1. The top selected model is given in bold.

| **Model ID** | **Model structure** | **K** | **AIC** | **ΔAIC** | ***w_i_*** | **Dev.** | **% Dev. Expl.** |
| --- | --- | --- | --- | --- | --- | --- | --- |
| H0 | 1 | 1 | 2343.3 | 1442.2 | 0.00 | 2341 | 0.0 |
| H1 | SDtribmn+SDtribmn^2^ | 3 | 1129.8 | 228.7 | 0.00 | 1124 | 52.0 |
| H2 | SDbrdlfbmn+SDmixedbmn+SDconifbmn | 4 | 1168.7 | 267.6 | 0.00 | 1161 | 50.4 |
| H3 | SDbrdlfbmn+SDmixedbmn+SDconifbmn+SDshrbmn | 5 | 1134.9 | 233.8 | 0.00 | 1125 | 51.9 |
| H4 | SDbrdlfbmn+SDconifbmn+SDtribmn+SDtribmn^2^ | 5 | 1038.8 | 137.7 | 0.00 | 1029 | 56.0 |
| H5 | SDpastbmn+SDpastbmn^2^+SDagricbmn+SDagricbmn^2^+SDartifbmn | 6 | 1073.0 | 171.9 | 0.00 | 1061 | 54.7 |
| H6 | SDnatrdbmn+SDnatrdbmn^2^+SDcourdbmn+SDcourdbmn^2^+SDcomrdbmn+SDcomrdbmn^2^ | 7 | 1713.5 | 812.4 | 0.00 | 1699 | 27.4 |
| H7 | SDnatrdbmn+SDnatrdbmn^2^+SDcourdbmn+SDcourdbmn^2^+SDcomrdbmn+SDcomrdbmn^2^+SDartifbmn | 8 | 1581.3 | 680.2 | 0.00 | 1565 | 33.1 |
| H8 | SDnatrdbmn+SDnatrdbmn^2^+SDcourdbmn+SDcourdbmn^2^+SDcomrdbmn+SDcomrdbmn^2^+SDpastbmn+SDpastbmn^2^+SDagricbmn+SDagricbmn^2^+SDartifbmn | 12 | 1010.9 | 109.8 | 0.00 | 986.9 | 57.8 |
| H9 | SDartifbmn+SDtribmn+SDtribmn^2^ | 4 | 1112.6 | 211.5 | 0.00 | 1105 | 52.8 |
| H10 | SDpastbmn+SDpastbmn^2^+SDartifbmn+SDtribmn+SDtribmn^2^ | 6 | 1054.8 | 153.7 | 0.00 | 1043 | 55.4 |
| H11 | SDnatrdbmn+SDnatrdbmn^2^+SDcourdbmn+SDcourdbmn^2^+SDcomrdbmn+SDcomrdbmn^2^+SDtribmn+SDtribmn^2^ | 9 | 1062.9 | 161.8 | 0.00 | 1045 | 55.4 |
| H12 | SDpastbmn+SDpastbmn^2^+SDartifbmn+SDnatrdbmn+SDnatrdbmn^2^+SDcourdbmn+SDcourdbmn^2^+SDcomrdbmn+SDcomrdbmn^2^+SDtribmn+SDtribmn^2^ | 12 | 1003.2 | 102.1 | 0.00 | 979.2 | 58.2 |
| H13 | SDbrdlfbmn+SDnatrdbmn+SDnatrdbmn^2^+SDcourdbmn+SDcourdbmn^2^+SDcomrdbmn+SDcomrdbmn^2^ | 8 | 1638.2 | 737.1 | 0.00 | 1622 | 30.7 |
| H14 | SDbrdlfbmn+SDmixedbmn+SDconifbmn+SDnatrdbmn+SDnatrdbmn^2^+SDcourdbmn+SDcourdbmn^2^+SDcomrdbmn+SDcomrdbmn^2^ | 10 | 1109.2 | 208.1 | 0.00 | 1089 | 53.5 |
| H15 | SDbrdlfbmn+SDmixedbmn+SDconifbmn+SDshrbmn+SDnatrdbmn+SDnatrdbmn^2^+SDcourdbmn+SDcourdbmn^2^+SDcomrdbmn+SDcomrdbmn^2^ | 11 | 1077.4 | 176.3 | 0.00 | 1055 | 54.9 |
| **H16** | **SDmixedbmn+SDconifbmn+SDnatrdbmn+SDnatrdbmn^2^+SDcourdbmn+SDcourdbmn^2^+SDcomrdbmn+SDcomrdbmn^2^+SDpastbmn+SDpastbmn^2^+SDagricbmn+SDagricbmn^2^+SDartifbmn** | **14** | **901.1** | **0.0** | **1.00** | **873.1** | **62.7** |
| H17 | SDbrdlfbmn+SDnatrdbmn+SDnatrdbmn^2^+SDcourdbmn+SDcourdbmn^2^+SDcomrdbmn+SDcomrdbmn^2^+SDtribmn+SDtribmn^2^ | 10 | 1004.0 | 102.9 | 0.00 | 984 | 58.0 |
| H18 | SDbrdlfbmn+SDconifbmn+SDnatrdbmn+SDnatrdbmn^2^+SDcourdbmn+SDcourdbmn^2^+SDcomrdbmn+SDcomrdbmn^2^+SDtribmn+SDtribmn^2^ | 11 | 989.3 | 88.2 | 0.00 | 967.3 | 58.7 |
| H19 | SDbrdlfbmn+SDnatrdbmn+SDnatrdbmn^2^+SDcourdbmn+SDcourdbmn^2^+SDcomrdbmn+SDcomrdbmn^2^+SDpastbmn+SDpastbmn^2^+SDartifbmn+SDtribmn+SDtribmn^2^ | 13 | 966.3 | 65.2 | 0.00 | 940.3 | 59.8 |
| H20 | SDbrdlfbmn+SDconifbmn+SDnatrdbmn+SDnatrdbmn^2^+SDcourdbmn+SDcourdbmn^2^+SDcomrdbmn+SDcomrdbmn^2^+SDpastbmn+SDpastbmn^2^+SDartifbmn+SDtribmn+SDtribmn^2^ | 14 | 945.5 | 44.4 | 0.00 | 917.5 | 60.8 |

H0: Null model

H1-H4: *Natural* habitat characteristics

H5-H8: *Human-generated* habitat features

H9-H12: *Natural* (abiotic) and *Human-generated* predictors;

H13-H16: *Natural* (biotic) and *Human-generated* variables

H17-H20: *Natural* (abiotic & biotic) and *Human-generated* variable combinations

K – number of parameters; AIC – Akaike’s Information Criterion; ΔAIC – difference in AIC between a given model and the corresponding null model; *w_i_* - Akaike weights; Dev. – Residual Deviance; % Dev. Expl. – Percentage of the Deviance Explained by a given model

Table S3. Candidate model set for gray wolf occurrence in Romania. Variable codes listed under ‘Model structure’ are provided in Table 1. The top selected model is given in bold.

| **Model ID** | **Model structure** | **K** | **AIC** | **ΔAIC** | ***w_i_*** | **Dev.** | **% Dev. Expl.** |
| --- | --- | --- | --- | --- | --- | --- | --- |
| H0 | 1 | 1 | 2795.1 | 1718.0 | 0.00 | 2793 | 0.0 |
| H1 | SDtriwmn+SDtriwmn^2^ | 3 | 1256.6 | 179.5 | 0.00 | 1251 | 55.2 |
| H2 | SDbrdlfwmn+SDmixedwmn+SDconifwmn | 4 | 1440.6 | 363.5 | 0.00 | 1433 | 48.7 |
| H3 | SDbrdlfwmn+SDmixedwmn+SDconifwmn+SDshrwmn | 5 | 1424.4 | 347.3 | 0.00 | 1414 | 49.4 |
| H4 | SDbrdlfwmn+SDconifwmn+SDtriwmn+SDtriwmn^2^ | 5 | 1206.3 | 129.2 | 0.00 | 1196 | 57.2 |
| H5 | SDpastwmn+SDpastwmn^2^+SDagricwmn+SDagricwmn^2^+SDartifwmn | 6 | 1203.0 | 125.9 | 0.00 | 1191 | 57.4 |
| H6 | SDnatrdwmn+SDnatrdwmn^2^+SDcourdwmn+SDcourdwmn^2^+SDcomrdwmn+SDcomrdwmn^2^ | 7 | 2290.0 | 1212.9 | 0.00 | 2276 | 18.5 |
| H7 | SDnatrdwmn+SDnatrdwmn^2^+SDcourdwmn+SDcourdwmn^2^+SDcomrdwmn+SDcomrdwmn^2^+SDartifwmn | 8 | 2135.7 | 1058.6 | 0.00 | 2120 | 24.1 |
| H8 | SDnatrdwmn+SDnatrdwmn^2^+SDcourdwmn+SDcourdwmn^2^+SDcomrdwmn+SDcomrdwmn^2^+SDpastwmn+SDpastwmn^2^+SDagricwmn+SDagricwmn^2^+SDartifwmn | 12 | 1181.8 | 104.7 | 0.00 | 1158 | 58.5 |
| H9 | SDartifwmn+SDtriwmn+SDtriwmn^2^ | 4 | 1237.5 | 160.4 | 0.00 | 1229 | 56.0 |
| H10 | SDpastwmn+SDpastwmn^2^+SDartifwmn+SDtriwmn+SDtriwmn^2^ | 6 | 1162.6 | 85.5 | 0.00 | 1151 | 58.8 |
| H11 | SDnatrdwmn+SDnatrdwmn^2^+SDcourdwmn+SDcourdwmn^2^+SDcomrdwmn+SDcomrdwmn^2^+SDtriwmn+SDtriwmn^2^ | 9 | 1189.6 | 112.5 | 0.00 | 1172 | 58.0 |
| H12 | SDpastwmn+SDpastwmn^2^+SDartifwmn+SDnatrdwmn+SDnatrdwmn^2^+SDcourdwmn+SDcourdwmn^2^+SDcomrdwmn+SDcomrdwmn^2^+SDtriwmn+SDtriwmn^2^ | 12 | 1125.4 | 48.3 | 0.00 | 1101 | 60.6 |
| H13 | SDbrdlfwmn+SDnatrdwmn+SDnatrdwmn^2^+SDcourdwmn+SDcourdwmn2+SDcomrdwmn+SDcomrdwmn^2^ | 8 | 1949.1 | 872.0 | 0.00 | 1933 | 30.8 |
| H14 | SDbrdlfwmn+SDmixedwmn+SDconifwmn+SDnatrdwmn+SDnatrdwmn^2^+SDcourdwmn+SDcourdwmn^2^+SDcomrdwmn+SDcomrdwmn^2^ | 10 | 1369.6 | 292.5 | 0.00 | 1350 | 51.7 |
| H15 | SDbrdlfwmn+SDmixedwmn+SDconifwmn+SDshrwmn+SDnatrdwmn+SDnatrdwmn^2^+SDcourdwmn+SDcourdwmn^2^+SDcomrdwmn+SDcomrdwmn^2^ | 11 | 1355.8 | 278.7 | 0.00 | 1334 | 52.2 |
| H16 | SDmixedwmn+SDconifwmn+SDnatrdwmn+SDnatrdwmn^2^+SDcourdwmn+SDcourdwmn^2^+SDcomrdwmn+SDcomrdwmn^2^+SDpastwmn+SDpastwmn^2^+SDagricwmn+SDagricwmn^2^+SDartifwmn | 14 | 1120.5 | 43.4 | 0.00 | 1093 | 60.9 |
| H17 | SDbrdlfwmn+SDnatrdwmn+SDnatrdwmn^2^+SDcourdwmn+SDcourdwmn^2^+SDcomrdwmn+SDcomrdwmn^2^+SDtriwmn+SDtriwmn^2^ | 10 | 1177.2 | 100.1 | 0.00 | 1157 | 58.6 |
| H18 | SDbrdlfwmn+SDconifwmn+SDnatrdwmn+SDnatrdwmn^2^+SDcourdwmn+SDcourdwmn^2^+SDcomrdwmn+SDcomrdwmn^2^+SDtriwmn+SDtriwmn^2^ | 11 | 1141.9 | 64.8 | 0.00 | 1120 | 59.9 |
| H19 | SDbrdlfwmn+SDnatrdwmn+SDnatrdwmn^2^+SDcourdwmn+SDcourdwmn^2^+SDcomrdwmn+SDcomrdwmn^2^+SDpastwmn+SDpastwmn^2^+SDartifwmn+SDtriwmn+SDtriwmn^2^ | 13 | 1126.6 | 49.5 | 0.00 | 1101 | 60.6 |
| **H20** | **SDbrdlfwmn+SDconifwmn+SDnatrdwmn+SDnatrdwmn^2^+SDcourdwmn+SDcourdwmn^2^+SDcomrdwmn+SDcomrdwmn^2^+SDpastwmn+SDpastwmn^2^+SDartifwmn+SDtriwmn+SDtriwmn^2^** | **14** | **1077.1** | **0.0** | **1.00** | **1049** | **62.4** |

H0: Null model

H1-H4: *Natural* habitat characteristics

H5-H8: *Human-generated* habitat features

H9-H12: *Natural* (abiotic) and *Human-generated* predictors;

H13-H16: *Natural* (biotic) and *Human-generated* variables

H17-H20: *Natural* (abiotic & biotic) and *Human-generated* variable combinations

K – number of parameters; AIC – Akaike’s Information Criterion; ΔAIC – difference in AIC between a given model and the corresponding null model; *w_i_* - Akaike weights; Dev. – Residual Deviance; % Dev. Expl. – Percentage of the Deviance Explained by a given model

Table S4. Candidate model set for Eurasian lynx occurrence in Romania. Variable codes listed under ‘Model structure’ are provided in Table 1. The top selected model is given in bold.

| **Model ID** | **Model structure** | **K** | **AIC** | **ΔAIC** | ***w_i_*** | **Dev.** | **% Dev. Expl.** |
| --- | --- | --- | --- | --- | --- | --- | --- |
| H0 | 1 | 1 | 2395.0 | 1702.1 | 0.00 | 2393 | 0.0 |
| H1 | SDtrilmn+SDtrilmn^2^ | 3 | 802.4 | 109.5 | 0.00 | 796.4 | 66.7 |
| H2 | SDbrdlflmn+SDmixedlmn+SDconiflmn | 4 | 845.9 | 153.0 | 0.00 | 837.9 | 65.0 |
| H3 | SDbrdlflmn+SDmixedlmn+SDconiflmn+SDshrlmn | 5 | 834.1 | 141.2 | 0.00 | 824.1 | 65.6 |
| H4 | SDbrdlflmn+SDconiflmn+SDtrilmn+SDtrilmn^2^ | 5 | 752.8 | 59.9 | 0.00 | 742.8 | 69.0 |
| H5 | SDpastlmn+SDpastlmn^2^+SDagriclmn+SDagriclmn^2^+SDartiflmn | 6 | 818.8 | 125.9 | 0.00 | 806.8 | 66.3 |
| H6 | SDnatrdlmn+SDnatrdlmn^2^+SDcourdlmn+SDcourdlmn^2^+SDcomrdlmn+SDcomrdlmn^2^ | 7 | 1557.5 | 864.6 | 0.00 | 1543 | 35.5 |
| H7 | SDnatrdlmn+SDnatrdlmn^2^+SDcourdlmn+SDcourdlmn^2^+SDcomrdlmn+SDcomrdlmn^2^+SDartiflmn | 8 | 1436.9 | 744.0 | 0.00 | 1421 | 40.6 |
| H8 | SDnatrdlmn+SDnatrdlmn^2^+SDcourdlmn+SDcourdlmn^2^+SDcomrdlmn+SDcomrdlmn^2^+SDpastlmn+SDpastlmn^2^+SDagriclmn+SDagriclmn^2^+SDartiflmn | 12 | 734.2 | 41.3 | 0.00 | 710.2 | 70.3 |
| H9 | SDartiflmn+SDtrilmn+SDtrilmn^2^ | 4 | 803.0 | 110.0 | 0.00 | 794.9 | 66.8 |
| H10 | SDpastlmn+SDpastlmn^2^+SDartiflmn+SDtrilmn+SDtrilmn^2^ | 6 | 805.9 | 112.9 | 0.00 | 793.8 | 66.8 |
| H11 | SDnatrdlmn+SDnatrdlmn^2^+SDcourdlmn+SDcourdlmn^2^+SDcomrdlmn+SDcomrdlmn^2^+SDtrilmn+SDtrilmn^2^ | 9 | 717.4 | 24.5 | 0.00 | 699.4 | 70.8 |
| H12 | SDpastlmn+SDpastlmn^2^+SDartiflmn+SDnatrdlmn+SDnatrdlmn^2^+SDcourdlmn+SDcourdlmn^2^+SDcomrdlmn+SDcomrdlmn^2^+SDtrilmn+SDtrilmn^2^ | 12 | 722.2 | 29.3 | 0.00 | 698.2 | 70.8 |
| H13 | SDbrdlflmn+SDnatrdlmn+SDnatrdlmn^2^+SDcourdlmn+SDcourdlmn^2^+SDcomrdlmn+SDcomrdlmn^2^ | 8 | 1325.5 | 632.6 | 0.00 | 1309 | 45.3 |
| H14 | SDbrdlflmn+SDmixedlmn+SDconiflmn+SDnatrdlmn+SDnatrdlmn^2^+SDcourdlmn+SDcourdlmn^2^+SDcomrdlmn+SDcomrdlmn^2^ | 10 | 767.3 | 74.3 | 0.00 | 747.3 | 68.8 |
| H15 | SDbrdlflmn+SDmixedlmn+SDconiflmn+SDshrlmn+SDnatrdlmn+SDnatrdlmn^2^+SDcourdlmn+SDcourdlmn^2^+SDcomrdlmn+SDcomrdlmn^2^ | 11 | 753.3 | 60.3 | 0.00 | 731.3 | 69.4 |
| **H16** | **SDmixedlmn+SDconiflmn+SDnatrdlmn+SDnatrdlmn^2^+SDcourdlmn+SDcourdlmn^2^+SDcomrdlmn+SDcomrdlmn^2^+SDpastlmn+SDpastlmn^2^+SDagriclmn+SDagriclmn^2^+SDartiflmn** | **14** | **692.9** | **0.0** | **0.99** | **664.9** | **72.2** |
| H17 | SDbrdlflmn+SDnatrdlmn+SDnatrdlmn^2^+SDcourdlmn+SDcourdlmn^2^+SDcomrdlmn+SDcomrdlmn^2^+SDtrilmn+SDtrilmn^2^ | 10 | 716.6 | 23.7 | 0.00 | 696.6 | 70.9 |
| H18 | SDbrdlflmn+SDconiflmn+SDnatrdlmn+SDnatrdlmn^2^+SDcourdlmn+SDcourdlmn^2^+SDcomrdlmn+SDcomrdlmn^2^+SDtrilmn+SDtrilmn^2^ | 11 | 702.7 | 9.8 | 0.01 | 680.7 | 71.6 |
| H19 | SDbrdlflmn+SDnatrdlmn+SDnatrdlmn^2^+SDcourdlmn+SDcourdlmn^2^+SDcomrdlmn+SDcomrdlmn^2^+SDpastlmn+SDpastlmn^2^+SDartiflmn+SDtrilmn+SDtrilmn^2^ | 13 | 722.1 | 29.2 | 0.00 | 696.1 | 70.9 |
| H20 | SDbrdlflmn+SDconiflmn+SDnatrdlmn+SDnatrdlmn^2^+SDcourdlmn+SDcourdlmn^2^+SDcomrdlmn+SDcomrdlmn^2^+SDpastlmn+SDpastlmn^2^+SDartiflmn+SDtrilmn+SDtrilmn^2^ | 14 | 707.2 | 14.3 | 0.00 | 679.2 | 71.6 |

H0: Null model

H1-H4: *Natural* habitat characteristics

H5-H8: *Human-generated* habitat features

H9-H12: *Natural* (abiotic) and *Human-generated* predictors;

H13-H16: *Natural* (biotic) and *Human-generated* variables

H17-H20: *Natural* (abiotic & biotic) and *Human-generated* variable combinations

K – number of parameters; AIC – Akaike’s Information Criterion; ΔAIC – difference in AIC between a given model and the corresponding null model; *w_i_* - Akaike weights; Dev. – Residual Deviance; % Dev. Expl. – Percentage of the Deviance Explained by a given model


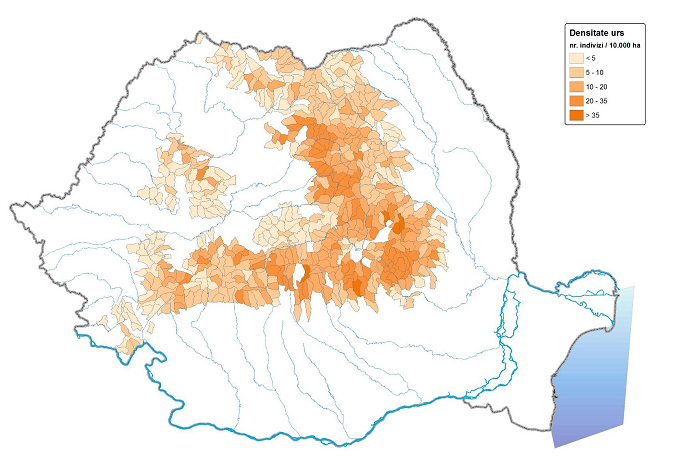


Fig. S1 Map No. 6 – Density of the bear population estimated for the 2011-2012 hunting season, at the level of WMUs. Reproduced with permission from Jurj and Ionescu 2011. Darkening shades of brown indicate increasing bear densities (individuals per 10,000 ha). WMUs with no bears present based on snow tracking data are not illustrated.


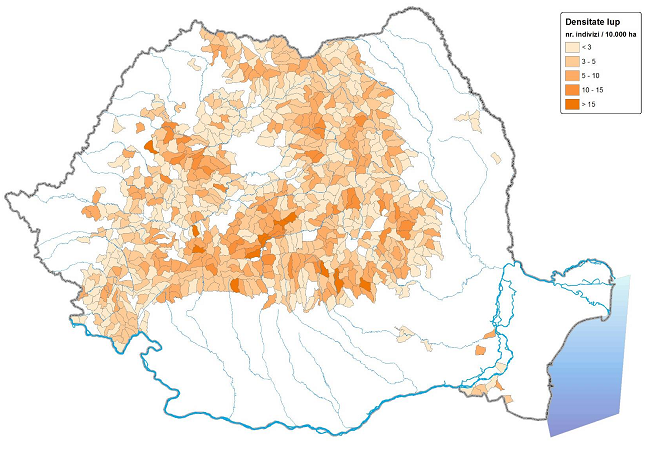


Fig. S2 Map No. 8 – Density of the wolf population estimated for the 2011-2012 hunting season, at the level of WMUs. Reproduced with permission from Jurj and Ionescu 2011. Darkening shades of brown indicate increasing wolf densities (individuals per 10,000 ha). WMUs with no wolves present based on snow tracking data are not illustrated.


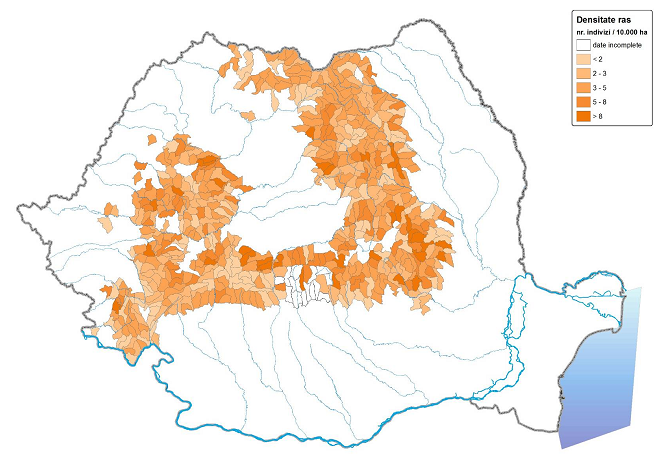


Fig. S3 Map No. 10 – Density of the lynx population estimated for the 2011-2012 hunting season, at the level of WMUs. Reproduced with permission from Jurj and Ionescu 2011. Darkening shades of brown indicate increasing lynx densities (individuals per 10,000 ha). WMUs with no lynx present based on snow tracking data are not illustrated.

**Supplementary literature cited**

EIONET. 2013. European Topic Centre on Spatial Information and Analyis: Corine Land Cover. http://sia.eionet.europa.eu/CLC2000/classes/index_html. Accessed 1 November 2014.

Jurj R, Ionescu O. 2011. Raport final pentru „Studiul privind estimarea populaţiilor de carnivore mari şi pisică sălbatică din România (*Ursus arctos*, *Canis lupus*, *Lynx lynx* şi *Felis silvestris*) în vederea menţinerii într-o stare favorabilă de conservare şi pentru stabilirea numărului de exemplare din speciile strict protejate care se pot recolta în cadrul sezonului de vânătoare 2011-2012”. [In Romanian] Final report for “Study on estimating large carnivore and wildcat populations in Romania (*Ursus arctos*, *Canis lupus*, *Lynx lynx* and *Felis silvestris*) for maintaining favorable conservation status and for delineating the numbers of specimens from strictly protected species which can be harvested during the 2011-2012 hunting season”. Fundatia Carpati, Institutul de Cercetari si Amenajari Silvice, Universitatea “Transilvania” din Brasov

Online: http://www.mmediu.ro/protectia_naturii/biodiversitate/carnivore_mari/2012-01-16/2012-01-16_carnivore_mari_evaluare_2011_raportcarnivoremari2011.pdf. Accessed: 1.08.2014.
